# Study on the Powder-Spreading Process of Walnut Shell/Co-PES Biomass Composite Powder in Additive Manufacturing

**DOI:** 10.3390/ma16124295

**Published:** 2023-06-09

**Authors:** Yueqiang Yu, Tingang Ma, Suling Wang, Minzheng Jiang, Sheng Gao, Yanling Guo, Ting Jiang, Bakary S. Doumbia, Bo Yan, Shaorui Shen

**Affiliations:** 1 College of Mechanical Science and Engineering, Northeast Petroleum University, Daqing 163318, China; yuyaoqiang.1228@163.com (Y.Y.); mtinga0@163.com (T.M.); jmz_1963@163.com (M.J.); jiangting1112@163.com (T.J.); 18710605661@163.com (B.Y.); ssr897419540@163.com (S.S.); 2 Key Laboratory of Petroleum Mechanical Engineering of Heilongjiang Province, Daqing 163318, China; 3 Research and Development Center of 3D Printing Material and Technology, Northeast Forestry University, Harbin 150040, China; nefugyl@hotmail.com; 4 Forestry and Woodworking Machinery Engineering Technology Center, Northeast Forestry University, Harbin 150040, China; doumbiabs@126.com

**Keywords:** additive manufacturing, powder laying, discrete element method, biomass composite powder

## Abstract

Powder laying is a necessary procedure during powder bed additive manufacturing (PBAM), and the quality of powder bed has an important effect on the performance of products. Because the powder particle motion state during the powder laying process of biomass composites is difficult to observe, and the influence of the powder laying process parameters on the quality of the powder bed is still unclear, a simulation study of the biomass composite powder laying process during powder bed additive manufacturing was conducted using the discrete element method. A discrete element model of walnut shell/Co-PES composite powder was established using the multi-sphere unit method, and the powder-spreading process was numerically simulated using two different powder spreading methods (rollers/scrapers). The results showed that the quality of powder bed formed by roller laying was better than that formed by scrapers with the same powder laying speed and powder laying thickness. For both of the two different spreading methods, the uniformity and density of the powder bed decreased as spreading speed increased, although the spreading speed had a more important influence on scraper spreading compared to roller spreading. As powder laying thickness increased, the powder bed formed by the two different powder laying methods became more uniform and denser. When the powder laying thickness was less than 110μm, the particles were easily blocked at the powder laying gap and are pushed out of the forming platform, forming many voids, and decreasing the powder bed’s quality. When the powder thickness was greater than 140 μm, the uniformity and density of the powder bed increased gradually, the number of voids decreased, and the quality of the powder bed improved.

## 1. Introduction

Powder Bed Additive Manufacturing (PBAM) uses 3D data to manufacture 3D parts by stacking materials layer by layer [1,2]. Compared with traditional removal material processing techniques, PBAM technology has the advantages of forming complex structural parts, short cycle time, and high material utilization [3], and can be divided into selective laser sintering (SLS), selective laser melting (SLM), and electron beam selective melting (EBSM) processes, depending on the laser energy and the material. Biomass composites are very promising green materials that have started to be used in additive manufacturing (AM) in the past few years. For example, Guo et al. [4] proposed the idea of using biomass composites for selective laser sintering and pointed out that biomass composites have the advantages of low cost, low power consumption, and reusability. Zhao et al. [5] prepared bamboo powder/co-polyamide composite powder and obtained molded parts with high molding accuracy by adjusting the process parameters. Zeng et al. [6] prepared rice husk composite powder with a mechanical mixing method, using rice husk powder as raw material, and performed laser sintering tests to obtain rice husk composite-powder-molded parts with sufficient strength and high dimensional accuracy. Yu et al. [7,8,9,10] systematically studied the feasibility of walnut shell/co-polyester hot melt (Co-PES) composite powder as a raw material for selective laser sintering. The density of the sintered walnut shell/Co-PES composite powder parts after the waxing post-treatment was significantly increased, and the mechanical properties were also improved. 

PBAM consists of two main components: powder paving and laser sintering [11]. First, the powder feedstock is laid on top of the substrate with a layer of powder under the action of a roller or a scraper, and then a high-energy heat source selectively sears or melts the target area based on 3D data, repeating these two stages to complete the molding of the target part. To improve the performance of molded parts, laser sintering has been extensively studied. [12,13,14]. In contrast, few studies can be found in the literature on the link with powder paving; the quality of the powder bed formed during the lay-up process will have an important impact on the subsequent laser sintering and quality of the formed product [15,16]. For example, Cao et al. [13] showed the impact of powder lay-up quality on selective laser melting single-pass forming, and they found that powder beds with high stacking densities had stable melt pools and low orbital width volatility. A uniform and dense powder bed is conducive to the formation of a continuous and stable melt pool trajectory, reduces spattering, spalling and other defects, reduces the porosity and surface roughness of the parts, and improves the final mechanical properties of the parts [12,17]. The quality of the powder bed is determined by the powder-spreading process [18]. Although biomass materials have started to be used in additive manufacturing, the research on the powder-spreading process of biomass materials is still relatively scarce. Therefore, to manufacture high-quality biomass composite products, it is necessary to study the powder lay-up process and the powder layers laid down for material composites.

The powder-spreading process is a typical discrete solid laying process, and the structure of the powder bed in terms of density, homogeneity, and internal distribution depends on the state of motion of each particle. Using the most advanced experimental methods, it is also difficult to study the state of motion of individual particles as well as the structure of particle buildup. In recent years, more and more attention has been paid to the discrete element method (DEM) in additive manufacturing. Deng et al. [19] investigated the influence of particle size, aspect ratio, and cohesion on the powder accumulation state using the DEM method. To obtain structural information such as bulk density, porosity, and coordination number, which are difficult to measure directly in experiments, Chen et al. [11] analyzed the influence of the friction coefficient of powder and particle size on its flow characteristics and the uniformity and compactness of powder layer. They found that particle sizes less than a certain critical value would increase cohesion and decrease powder mobility, while reducing the friction factor could effectively enhance powder mobility and form a more dense and uniform powder bed. Zhang et al. [20] simulated nylon powder laying with rollers and scrapers, and found that, in a thicker powder layer, a higher density powder layer could be obtained using roller-type powder laying due to the action of compaction. With the increase in powder laying thickness, roller-type powder laying is more sensitive to the Segregation index than scraper-type powder spreading. Yao et al. [21] found that, during 316 L stainless steel powder spreading, a slower scraper movement speed means a better powder bed quality, but a lower efficiency. Chen et al. [22] studied the process of scraper powder laying by combining experiments and computational models, and determined three deposition mechanisms that dominated the powder diffusion process: cohesion effect, wall effect, and penetration effect.

However, previous studies have mostly focused on metal and polymer powders, and relatively little research has been conducted on biomass composite powders. To improve the quality of biomass composite parts, further research on their powder paving process is needed. In this paper, we innovatively used the DEM to analyze the powder-spreading process of biomass composites. Compared with biomass materials such as wood, bamboo, and rice husk, walnut shells are easy to process and crush. The particle size range required for powder bed additive manufacturing can be easily reached, and the crushed particles are nearly spherical in shape, which means they can achieve a good powder-spreading effect [8]. In our group’s previous study [7], we successfully realized the molding of walnut shell/co-polyester hot melt adhesive (Co-PES) parts using SLS technology, but further research on its powder-spreading mechanism is needed. Therefore, walnut shell/co-polyester hot melt adhesive (Co-PES) powder was used as the research object and the discrete element model of walnut shell/Co-PES powder was established using the multi-sphere unit method, and the powder-spreading process was numerically simulated. The density and uniformity of the powder bed were quantitatively determined using mathematical statistics, and the effects of laying speed, powder laying thickness, and different spreading methods (rollers/scrapers) on the uniformity and density of the powder bed were discussed and analyzed. This study provides a theoretical basis for the application of biomass composite powders in PBAM.

## 2. Computational Models

The DEM was first proposed by Dr. Peter Cundall in 1971, when he was studying for his PhD at Imperial College, University of London [23]. In the DEM simulation, each particle is modeled as an independent unit and simulated. By analyzing the state information of each cell, the motion law of the whole object can be obtained [24]. Currently, the common DEMs include the hard-sphere method and the soft-sphere method. In the hard-sphere model, the interaction between particles is controlled by the law of conservation of momentum, and the details of contact force and particle surface deformation are completely ignored. In the soft-sphere model, particles are considered as elastic objects, allowing some deformation, and the interaction between particles has a certain time, which can simulate the elastic–plastic contact forces, frictional forces and van der Waals forces between particles [25,26]. Since the soft-sphere model can realistically reflect the collision information between particles, the discrete element model of walnut shell/Co-PES powder will be established using the soft-sphere model.

**Figure 1:**
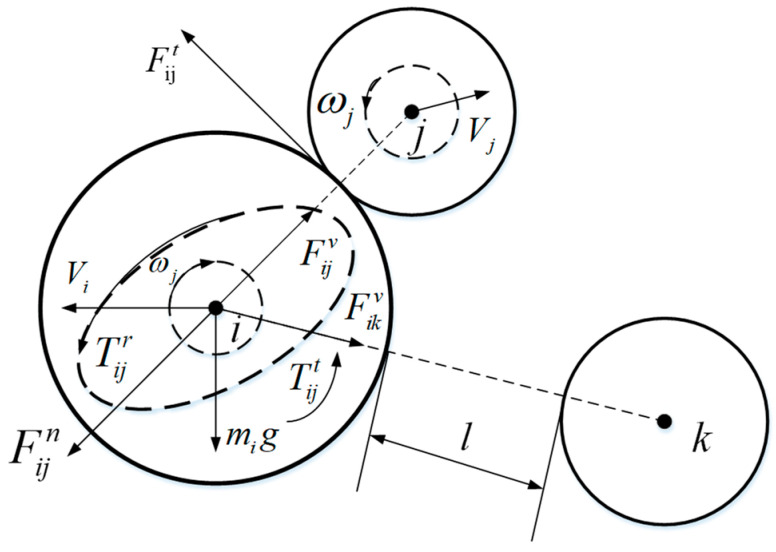
Plot of the effect of contacted particle j and uncontacted particle k on particle i.

The motion of particles follows Newton’s law of motion. Particle i has translational and rotational motion during the random filling process, and particles are affected by contact force, non-contact force and gravity, in Figure 1. The governing equation is:

(1) $$m_{i}\frac{d^{2}r_{i}}{dt^{2}}=\sum_{j}(F_{ij}^{n}+F_{ij}^{t}+F_{ij}^{v})+m_{i}g$$

(2) $$I_{i}\frac{dw_{i}}{dt}=\sum_{j}(T_{ij}^{t}+T_{ij}^{r})$$

where *m_i_* and *I_i_* are the mass and moment of inertia of particle i, respectively, and *r_i_* and *w_i_* are the displacement and angular velocity of particle i, respectively.

$F_{ij}^{n}\;and\;F_{ij}^{t}$ are the normal contact force and tangential contact force of particle j to particle i, respectively [27]. They can be calculated using the following formula:

(3) Fijn=[23ER¯δn32−γER¯δn(vij·nij)]nij

(4) $$F_{\mathrm{ij}}^{t}=\mu \left|F_{\mathrm{ij}}^{n}\right|\left[1-{\left(1-\frac{\left|\delta_{t}\right|}{\left|\delta_{\max}\right|}\right)}^{1.5}\right]t_{\mathrm{ij}}$$

(5) $$E=Y/(1-\sigma^{2})$$

(6) $$\bar{R}=R_{i}R_{j}/(R_{i}+R_{j})$$

(7) $$\delta_{n}=R_{i}+R_{j}-R_{ij}$$

(8) $$\delta_{t}=\int_{t_{0}}^{t}v_{\mathrm{ij}}^{t}dt$$

(9) $$\delta_{\max}=\mu \frac{2-\sigma }{2-2\sigma }\delta_{n}$$

(10) $$v_{\mathrm{ij}}=v_{i}-v_{j}-(R_{i}\omega_{i}\times n_{\mathrm{ij}}+R_{j}\omega_{i}\times n_{\mathrm{ij}})$$

(11) $$v_{\mathrm{ij}}^{t}=v_{\mathrm{ij}}-(v_{\mathrm{ij}}{\cdot n}_{\mathrm{ij}})n_{\mathrm{ij}}$$

(12) $$t_{\mathrm{ij}}=v_{\mathrm{ij}}^{t}/\left|v_{\mathrm{ij}}^{t}\right|$$

The meaning of the parameters represented in the above equation are presented in Table 1.

For the tangential contact force $F_{ij}^{t}$ between particles i and j, if $\left|F_{ij}^{t}\right|\geq \mu \left|F_{ij}^{n}\right|$, then $\left|F_{ij}^{t}\right|=-\mu \left|F_{ij}^{t}\right|t_{ij}$ [28].

$F_{ij}^{v}$ is the van der Waals force of particle j on particle i, calculated as follows:

(13) $$F_{\mathrm{ij}}^{v}=-\frac{\mathrm{Ha}}{6}\times \frac{64R_{i}^{3}R_{j}^{3}(l+R_{i}+R_{j})}{{\left(l^{2}+2R_{i}l+2R_{j}l\right)}^{2}{\left(l^{2}+2R_{i}l+2R_{j}l+4R_{i}R_{j}\right)}^{2}}n_{\mathrm{ij}}$$

where *Ha* is the Hamaker constant; *l* is the particle spacing; and the size is $R_{i}+R_{j}-R_{ij}$. When the distance between particles is too large or too small, Equation (13) has no practical meaning, so the range of *l* is limited to 1–100 nm. When beyond this range, it is considered that there is no van der Waals force between particles.

$T_{ij}^{t}\;and\;T_{ij}^{r}$ are the tangential contact force of particle j on particle i and the torque generated by rolling friction, respectively [27,29]. They are calculated as follows:

(14) $$T_{\mathrm{ij}}^{t}=-\mu R_{i}\left|F_{\mathrm{ij}}^{n}\right|\bar{\omega_{i}}$$

(15) $$T_{\mathrm{ij}}^{r}=R_{i}n_{\mathrm{ij}}\times T_{\mathrm{ij}}^{t}$$

In Equation (14), $\bar{\omega_{i}}$ is the unit vector; the calculation formula is $\bar{\omega_{i}}=\omega_{i}/\left|\omega_{i}\right|$.

Considering the effect of cohesion, using the Hertz–Mindlin JKR model, the normal cohesion is added in the contact zone as follows:

(16) FJKR=−4πγE*a32+4E*3R*a3

(17) $$\delta =\frac{a^{2}}{R^{*}}-\sqrt{\frac{4\pi \gamma \alpha }{E^{*}}}$$

where E* is the equivalent Young’s modulus, R* is the equivalent radius, *δ* is the overlap, and *γ* is the surface energy.

This model provides cohesive forces even without physical contact. Assuming that nonzero forces are present between particles, the following equation gives the maximum gap between them:

(18) $$\delta_{c}=-\sqrt{\frac{4\pi \gamma a_{c}}{E^{*}}}+\frac{a_{c}^{2}}{R^{*}}$$

(19) $$a_{c}={\left[\frac{9\pi \gamma R^{*2}}{2E^{*}}(\frac{3}{4}-\frac{1}{\sqrt{2}})\right]}^{\frac{1}{3}}$$

where *δ_c_* is the maximum gap between particles and *a_c_* is the maximum contact point radius.

The bonding force reaches its maximum when there is no physical contact between the particles and the particle distance is less than *i_ce_*. Its value is [30,31]:

(20) $$F_{pullout}=-\frac{3}{2}\pi \gamma R^{*}$$

**Figure 2:**
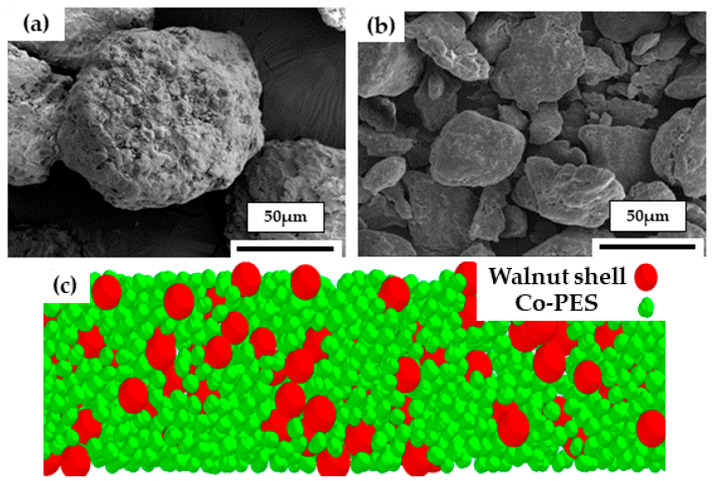
SEM image and computational model of powder; (**a**) Structure morphology of walnut shell powder particle; (**b**) Structure morphology of Co-PES powder particle; (**c**) Walnut shell/Co-PES composite powder analytical model.

At present, there are various DEM modeling methods for irregularly shaped particles. In this paper, the least computationally intensive multi-sphere unit method is used to model Co-PES composite powder. As shown in Figure 2a, the walnut shell particles are nearly spherical with a rough surface. Using a separate spherical element modeled as shown in Figure 2c, the diameter of the spherical element is the diameter of the walnut shell particle. The Co-PES powder particles are irregularly shaped with smooth particle surfaces, as seen in Figure 2b. In Figure 2c, the Co-PES powder was modeled as overlapping spherical units by the multi-sphere unit method, and the mass of Co-PES particles was obtained by Boolean subtraction operation. The breakage and fracture of Co-PES particles were not considered in this study. Walnut shell powder and Co-PES powder were mixed at a mass ratio of 1:4 in Figure 2c.

**Figure 3:**
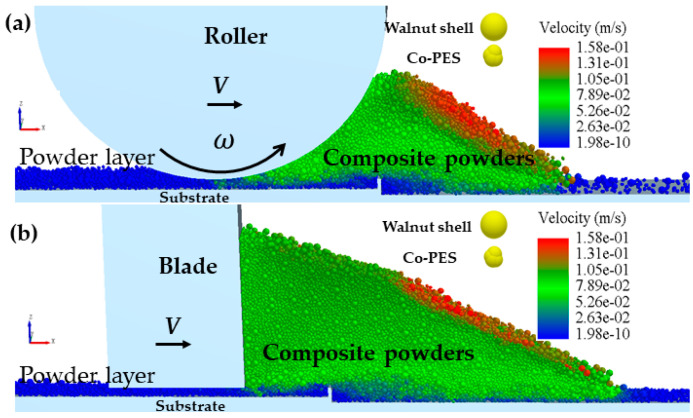
Physical model of two different ways of spreading powder; (**a**) roller spreading powder; (**b**) scraper spreading powder.

The model parameters of walnut shell and Co-PES powder used in the simulation are shown in Table 2. In the simulation, the geometry of the rollers and scrapers and the spreading of the powder are consistent with reality. Because of the smooth surface of the roller and the scraper, the friction coefficients of the rollers/scraper–particles (walnut shell/Co-PES) were set to be half of those of walnut shell–walnut shell and Co-PES–Co-PES. Because the substrate surface is rough, the substrate–particle (walnut shell/Co-PES) friction coefficient are set to twice the friction coefficients of walnut shell–walnut shell and Co-PES–Co-PES, respectively. In addition, in order that the motion of the roller and the scraper is not interfered, their masses are assumed to be infinite. To save calculation time, in Figure 3, the computational domain in the Y-direction for both powder layer processes was 500 μm with periodic boundaries. The Young’s modulus has little effect on the simulation results [32], and the Young’s modulus used in the simulations is two orders of magnitude smaller than the real value and, therefore, the surface energy density is also two orders of magnitude smaller than the real value to assure the same ratio between Young’s modulus and Hamaker’s constant and thus obtain reliable van der Waals forces [33]. The particles in the simulations were generated using a normal distribution in order to be practically equivalent.

## 3. Characterization of Powder Bed Density

**Figure 4:**
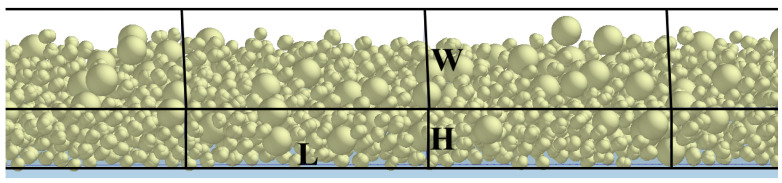
Grid size.

The effect of different parameters on the quality of the powder bed was investigated by analyzing the density of the powder bed. A series of grids with dimensions L × W × H were set up on the powder bed along the direction of the powder laying, as shown in Figure 4.

Define the average scores $\overset{-}{\rho }$ and the average scores standard deviation S of the grid to characterize the density of the powder bed and the uniformity of the powder bed density, respectively. The density calculation of the powder bed in the i-th grid is given as:

(21) $$\rho_{i}=\frac{m_{i}}{L\times W\times H}$$

where *m_i_* is the mass of the particle in the i-th grid.

The average scores of the grids are:

(22) $$\bar{\rho }=\frac{\sum_{i=1}^{N}\rho_{i}}{N}$$

where *N* is the number of grids.

The average score standard deviation *S* of the grid is:

(23) $$S=\sqrt{\frac{\sum_{i=1}^{N}{\left(\rho_{i}-\bar{\rho }\right)}^{2}}{N-1}}$$

## 4. Results and Discussion

### 4.1. Influence of Squeegee and Roller Spreading Powder

**Figure 5:**
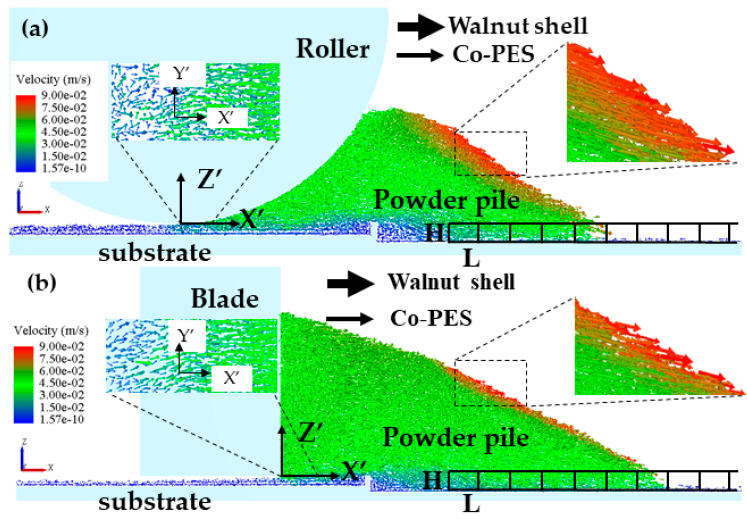
(**a**) Distribution of roller spreading speed; (**b**) Distribution of scraper spreading speed.

Scraper [36] and roller [37] spreading are commonly used in industrial equipment, but the spreading mechanisms of these two spreading methods are different, so their effects on the walnut shell/Co-PES hybrid powder bed need to be investigated. The results of the velocity field simulation of the walnut shell/Co-PES hybrid powder for the two powder laying methods are shown in Figure 5. The particle velocities are represented in absolute coordinates XYZ, and the motion coordinate system X′Y′Z′ is set at the bottom of the powder paver (rollers/scrapers) in order to be used to describe the dynamic behavior.

**Figure 6:**
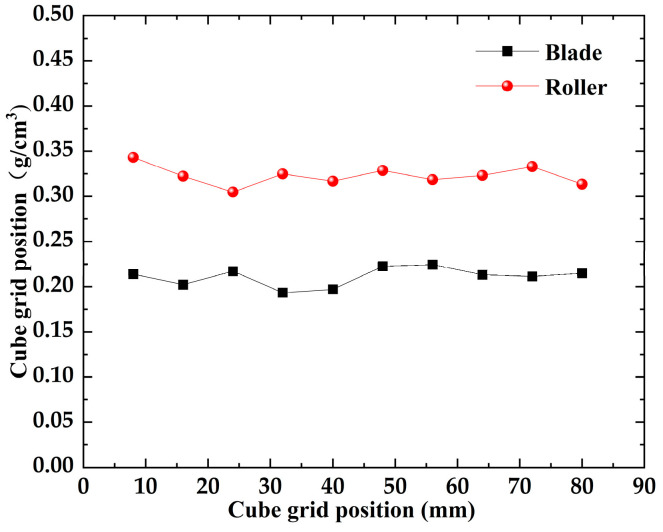
Cube mesh density.

In Figure 5a,b, for the particle velocity field, the mixed powder pile is pushed forward by the scraper/roller in the powder laying direction, and the powder pile upper layer particles accelerate and slide down under the effect of gravity, with a maximum velocity. For this reason, a large amount of kinetic energy is required to overcome the adhesion force between the powder particles, which can easily fall into the forming platform, and a powder layer is formed in the space between the scraper/roller and the substrate. An enlarged view is shown on the left of Figure 5a,b; there is some lateral motion in the YY′ direction due to random collisions, but the lateral movement of the powder pile can be neglected compared with the direction of powder laying, and we focus on the motion of the particles in the powder laydown direction. A plurality of rectangular grids with dimensions L × W × H are arranged along the X direction on the powder bed and used to measure the particle mass. The density of each cubic grid was calculated for the two different powder laying methods, as shown in Figure 6. Compared with the scraper spreading method, the roller spreading powder density is greater in each cube area and the formed powder layer is denser. This is because the effective contact area of the roller with the particles during the powder laying process is larger than that of the scraper with the powder, which facilitates the rearrangement of the particles. The scraper interacts with the powder layer mainly at its edge and drags the particles as it moves, resulting in a greater roughness. In addition, in the process of powder laying by reverse rotation of the roller, the roller will have a compacting effect on the powder bed, and the small particles will fill the space between the coarse particles, resulting in a higher mass of mixed powder under the same volume, more powder will be laid, and a denser powder layer will be formed [38]. Therefore, the powder layer formed by the roller is denser than that formed by the scraper.

### 4.2. Influence of Powder Laying Speed on Powder Bed

**Figure 7:**
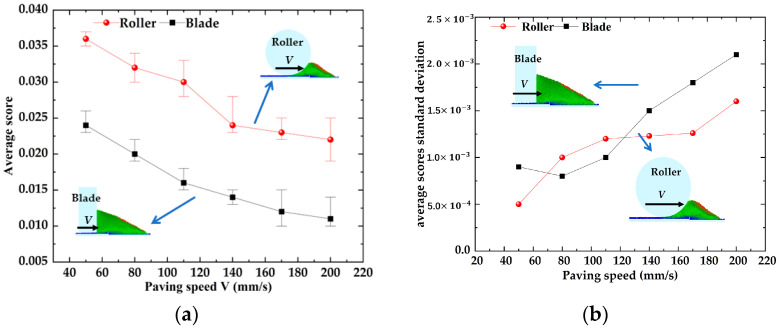
Influence of powder laying speed on powder bed. (**a**) Influence of powder laying speed on the average score of powder bed; (**b**) Influence of powder laying speed on the average scores standard deviation of the powder bed.

Increasing the powder spreading speed is usually considered an important method to improve the production efficiency [39], but the increase in powder spreading speed will have an impact on the quality of the powder bed, so the relationship between powder spreading speed and powder bed quality and the reasonable selection of powder spreading speed need to be studied. The effect of the powder laying speed on the average scores and their standard deviation of the powder bed in Figure 7. With an increase in powder laying speed, the average score of the bed decreases linearly, and the average score of the bed decreases from 0.036 to 0.022. The average scores of the scraper bed decreased from 0.024 to 0.011. With the increase in powder laying speed, the average scores standard deviation of the roll/scraper-formed powder bed showed an increasing trend. The average score standard deviation of the roll increased from 0.0005 to 0.0016, and that of the scraper increased from 0.0009 to 0.0021. The results showed that the faster the powder laying speed, the less powder is mixed into the bed, and the worse the uniformity and compactness of the bed.

**Figure 8:**
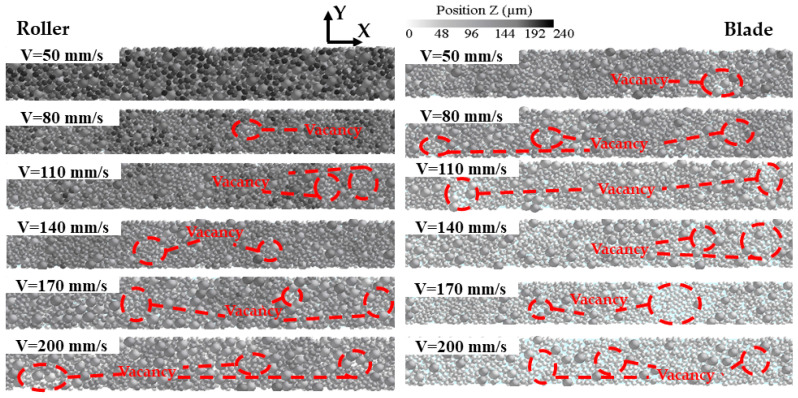
Simulation results of powder bed morphology under different powder laying speeds.

Figure 8 shows the effect of different spreading speeds on the morphology of the powder bed. For the two different powder laying methods, when the powder laying speed increases from 50 mm/s to 110 mm/s, a small vacancy gradually appears in the powder bed, making the density and uniformity of the powder bed decline. When the powder laying speed increases from 140 mm/s to 200 mm/s, the accumulation height of the powder bed declines rapidly and the vacancies in the powder bed become larger and larger, so the uniformity and compactness of the powder bed are reduced. In addition, increasing the powder laying speed has a great impact on the scraper, and the powder bed becomes very loose when the powder laying speed V > 140 mm/s, which also verifies the conclusion obtained from Figure 7.

**Figure 9:**
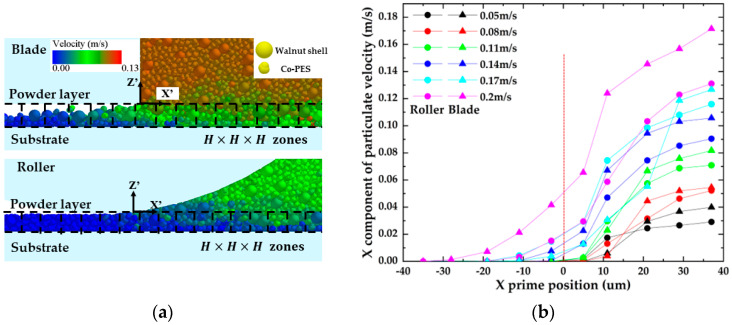
Effect of spreading speed on two forms of spreading: (**a**) H × H × H area division; (**b**) particle speed in H × H × H area.

To further investigate the effect of powder laying speed on the powder layer region, multiple cubic areas of size H × H × H are fixed along the direction of motion at the bottom of the roll/scraper to collect the average particle velocity in the X′ direction, as shown in Figure 9a. For both roller and scraper forms of powder spreading, as the speed of laying powder increases, the velocity of particles in the X′ direction in the cube area increases and particles move in the direction of X′, causing the powder bed to become loose and a decline in the powder bed quality. In addition, in the left area of the bottom of the roller/scraper, in Figure 9b, the particles will continue to move, and their speed increases with the increase in laying speed. This indicates that the mixed powder particles will still move on the substrate in the spreading direction after passing through the roller/scraper, which will make the powder bed looser. This is consistent with the conclusions in the literature [33]. This movement becomes more pronounced as the speed of powder laying increases, especially with the scraper.

From the above analysis, it is concluded that the uniformity and compactness of the powder bed decreased due to the high laying speed. However, in industrial production, the powder laying speed is usually increased to improve production efficiency, with the accompanying disadvantage of reducing the quality of the powder bed laying, which is detrimental to the production. Therefore, the maximum powder laying speed should be reasonably adjusted for both high-efficiency and high-quality production [40].

### 4.3. Influence of Powder Laying Thickness on Powder Bed

**Figure 10:**
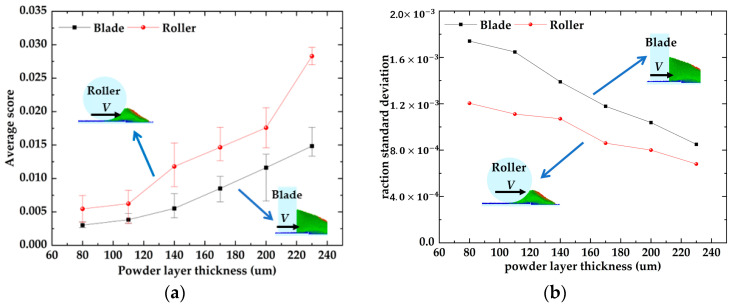
Influence of powder thickness on the density of powder bed. (**a**) Influence of powder thickness on the average score of powder bed; (**b**) Influence of powder thickness on the raction standard deviation of the powder bed.

The effects of powder laying thickness on the average scores and their standard deviations of the powder bed in Figure 10. The average scores of the powder bed formed by the two powder laying methods monotonically increase as the thickness of the powder layer increases, and the standard deviation of the average scores monotonically decline with increasing thickness of the powder layer. This indicates that the forming quality of the powder bed improved with increasing powder laying thickness. Furthermore, the monotonic trend of the average scores and average scores standard deviation became more and more remarkable with the gradual increase in the powder laying thickness. This implies that the forming quality of the powder bed improves faster as the powder laying thickness increases.

**Figure 11:**
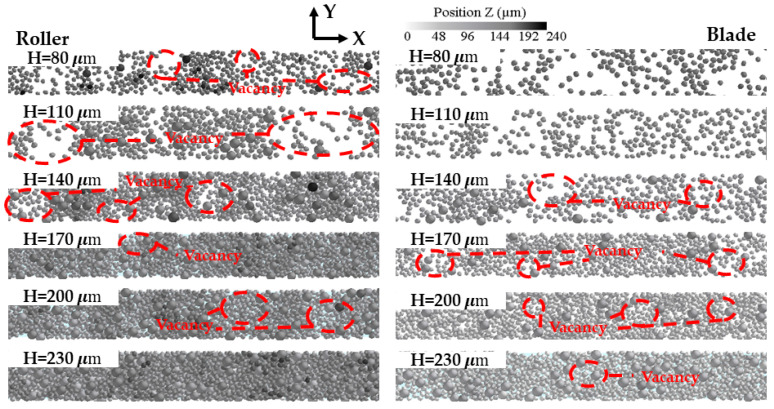
Simulation results of powder bed morphology under different powder laying thickness.

The powder bed morphology obtained from the DEM simulation more directly illustrates the effect of the powder laying thickness, as shown in Figure 11. For the two different powder laying methods, when the powder laying thickness was 80 μm and 110 μm, there were many vacancies in the powder bed, and the powder bed quality was poor. This is because when the powder laying thickness is too small, the particle clogging in the area in front of the roller and scraper becomes more enhanced. During particle clogging, a strong chain structure is formed between the particles, which makes the particles in the clogging state accumulate a large amount of strain energy in a short time. When the roller/scraper continues to move forward, the force chain structure formed during the blockage phase collapses, causing the strain energy to be rapidly released and transferred to the surrounding particles, which in turn causes these particles to fly out from the powder laying gap area [41] and be pushed out of the forming platform, resulting in a powder bed with many voids. When the powder laying thickness H ≥ 140 mm, most of the particles stay on the forming platform to form a powder bed, the powder bed voids become smaller and smaller, and the uniformity and density of the powder bed gradually increase. From these phenomena, it can be seen that increasing the thickness of powder laying can improve the uniformity and density of the powder bed.

## 5. Conclusions

The powder laying process of biomass composites in PBAM was simulated using a DEM. Walnut shell/Co-PES powder was used as the research object and the discrete element model of walnut shell/Co-PES powder was established using the multi-sphere unit method, and a typical roller and scraper spreading simulation was performed for the spreading process, and the effects of different spreading methods (rollers/scrapers), powder paving, and powder layer thickness on the uniformity and density of the powder bed were researched. The key findings are as follows:

(1) Under the same simulation conditions, the roller and scraper spreading process was simulated. Due to the compaction effect of rollers and particle rearrangement, the density of the powder bed formed by rollers was generally greater than that of formed by scrapers, especially with 30 mm of powder bed. The density of powder bed formed by rollers was 60% higher than that of powder bed formed by scrapers at the same position. Thus, a better quality of powder bed is obtained by rollers laying powder.
(2) For the two different powder laying methods, the average scores and the average score standard deviation of the powder bed density decreased and increased, respectively, with increasing powder laying speed. When the powder spreading speed was increased from 50 mm/s to 200 mm/s, the average fraction of the density of powder bed formed by rollers decreased by 38%, and that formed by scraper decreased by 54% This implies that the uniformity and density of the powder bed decreases with increasing powder laying speed and the density of the powder bed decreases more rapidly with scraper forming compared to roller forming.
(3) For the two different powder laying methods, the average scores, and the average score standard deviation of the powder bed density increased and decreased, respectively, with increasing powder layer thickness. Among them, the average fraction of the density of powder bed formed by rollers increased by 418% when the powder laying thickness increased from 80 μm to 230 μm, and that of scraper forming increased by 390%. This shows that the homogeneity and density of the powder bed increases with the increase in powder laying thickness. The density of the bed increases faster with the roller than that with the scraper.

The influence of different process parameters on the density of biomass composite powder bed was investigated with walnut shell/Co-PES powder. In this paper, the walnut shell particles are modeled as spherical. However, the walnut shell particles are approximately spherical but not truly spherical, and the spreading parameters are not optimized. Theoretically, the powder bed laying process of biomass composite powder is influenced by many other parameters, such as the moisture content of biomass powder, the composition ratio between biomass powder and matrix powder, and different rotation speeds of the rollers, etc., which were not studied in this paper due to the length limitation. In the future, a new model will be developed based on the real shape of walnut shells for process optimization and experimental validation of biomass composite powder parameters to provide reference for the application of biomass composite powder in powder bed additive manufacturing.

## Figures and Tables

**Table 1:** Formula symbols and meaning.

Symbol	Meaning
γ	Damping coefficient
σ	Poisson’s ratio of the particle material
Y	Young’s modulus of the particle material
μ	Coefficient of sliding friction between particles
$\delta_{n}$	Amount of normal deformation between particles i and j
$\delta_{t}$	Tangential deformation between particles i and j
$\delta_{max}$	Maximum allowed tangential deformation
$n_{ij}$	Unit vector from the spherical center of particle j to the spherical center of particle i
$\nu_{ij}$	Velocity of particle i relative to particle j at the contact point
$\nu_{ij}^{t}$	Tangential velocity of particle i relative to particle j at the contact point
$t_{ij}$	Tangential unit vector of particle i relative to particle j

**Table 2:** Model parameters used in the simulation.

		Walnut Shell	Co-PES	Walnut Shell/Co-PES
Material density	ρ (g/cm^3^)	0.48	0.7	0.686
Young’s modulus [34,35]	E (GPa)	13.1	7.56	0.6995
Poisson ratio [34,35]	ξ	0.29	0.4	0.35
Restitution coefficient	e	0.5	0.65	0.6
Sliding friction coefficient	$\mu_{s}$	0.7	0.55	0.65
Rolling friction coefficient	$\mu_{r}$	0.01	0.01	0.01
Surface energy density	$\lambda (\frac{mJ}{m^{2}})$			0.2
Diameter of roller	D (mm)	5		
Rotation speed of roller	ω (rad/s)	2π		
Paving speed	V (m/s)	0.05–0.2		
Layer thickness	H (μm)	80–230		

## Data Availability

Not applicable.
